# Misdiagnosis of pulmonary paragonimiasis as tuberculosis: A case report

**DOI:** 10.1097/MD.0000000000047555

**Published:** 2026-01-30

**Authors:** Rong Li, Xing Liu, Gao Song, Cai-Qiong Zhang, Ling-Jun Shen, Wei-Xian Li, Zhong-Ping Bai

**Affiliations:** a Department of Pharmacy, Pu’er People’s Hospital, Pu’er, China; b Department of Pharmacy, The Third People’s Hospital of Kunming/Yunnan Clinical Center for Infectious Diseases, Kunming, China.

**Keywords:** hemoptysis, metagenomic next-generation sequencing (mNGS), misdiagnosis, pulmonary paragonimiasis, pulmonary tuberculosis

## Abstract

**Rationale:**

Pulmonary paragonimiasis and pulmonary tuberculosis exhibit overlapping clinical and imaging manifestations, resulting in frequent misdiagnosis in endemic areas. This case underscores the value of metagenomic next-generation sequencing (mNGS) in correcting such misdiagnoses and emphasizes the importance of managing drug–drug interactions between antituberculosis agents and praziquantel.

**Patient concerns:**

An 18-year-old female from Yunnan, a paragonimiasis-endemic region, presented with recurrent cough, expectoration, and hemoptysis for 4 years. She was initially diagnosed with pulmonary tuberculosis based on a positive tuberculin pure protein derivative test and chest computed tomography findings but failed to respond to antituberculosis therapy.

**Diagnoses:**

Pulmonary paragonimiasis (initially misdiagnosed as pulmonary tuberculosis).

**Interventions:**

In-hospital tuberculosis-related tests (GeneXpert MTB/RIF, sputum/bronchoalveolar lavage fluid culture, bronchoscopic biopsy) were negative. Bronchoalveolar lavage fluid mNGS identified 87 Paragonimus sequences, and Paragonimus antibody enzyme-linked immunosorbent assay was positive. A history of raw crab ingestion was confirmed. Antituberculosis treatment was discontinued for 4 weeks (due to drug interaction), followed by oral praziquantel (1.2 g, 3 times daily for 3 consecutive days).

**Outcomes:**

Hemoptysis resolved within 15 days of treatment initiation, and peripheral blood parameters returned to normal ranges. Chest computed tomography at 2 months posttreatment showed marked reduction in lesion size, and complete resolution of pulmonary cavities was observed at the 6-month follow-up, with no recurrence of symptoms.

**Lessons:**

For chronic respiratory symptoms unresponsive to antituberculosis treatment in endemic regions, proactive inquiry of raw freshwater crustacean consumption history and combined use of serology/mNGS can improve diagnostic accuracy. A 4-week washout period after rifampicin discontinuation is critical before praziquantel administration.

## 1. Introduction

Paragonimiasis, also known as pulmonary dystomatosis or paragonimus infection, is a parasitic disease caused by Paragonimus through the ingestion of raw or undercooked crabs or crayfish. It is estimated that over 20 million people worldwide are infected with the disease and over 290 million people are at risk of infection.^[1]^ The clinical manifestations of this disease are diverse, insidious, and nonspecific, often leading to misdiagnosis or underdiagnosis. Pulmonary paragonimiasis can be misdiagnosed as pneumonia, bronchiectasis, tuberculosis, or lung cancer.^[2]^ Barennes et al demonstrated that ~2.5% of paragonimiasis cases are misdiagnosed as tuberculosis.^[3]^ In areas with high tuberculosis prevalence (e.g., Southwest China), ~25% (2/8) of adult paragonimiasis cases presenting with lung masses as the main manifestation were misdiagnosed as tuberculosis in Xishuangbanna, Yunnan, and ~3.3% (4/123) of pediatric paragonimiasis cases were co-infected with tuberculosis.^[4,5]^ In recent years, along with a shift in raw food consumption practices, the incidence of this disease has exhibited an upward trend in urban regions.^[6]^ Herein, we report a case of pulmonary paragonimiasis in a patient admitted to The Third People’s Hospital of Kunming, aiming to provide a reference for the clinical diagnosis and treatment of this disease.

## 2. Case presentation

An 18-year-old female student from Lincang City, Yunnan Province, China was admitted to the Third People’s Hospital of Kunming on September 9, 2022, with a chief complaint of recurrent cough, expectoration, and hemoptysis for 4 years. She reported no history of smoking, alcohol consumption, genetic disorders, known allergies, or recent travel. An out-of-hospital tuberculin pure protein derivative skin test was strongly positive (15 mm × 15 mm hard nodule), and a tuberculosis infection T-cell spot test showed positive results (antigen A: 200; antigen B: 200). Chest computed tomography (CT) at the same facility revealed patchy shadows in the right upper lobe, suggesting inflammatory exudation with bronchiectasis, leading to a preliminary diagnosis of tuberculosis. She was transferred to our hospital for specialized treatment due to inadequate response to prior interventions.

**Figure 1.**
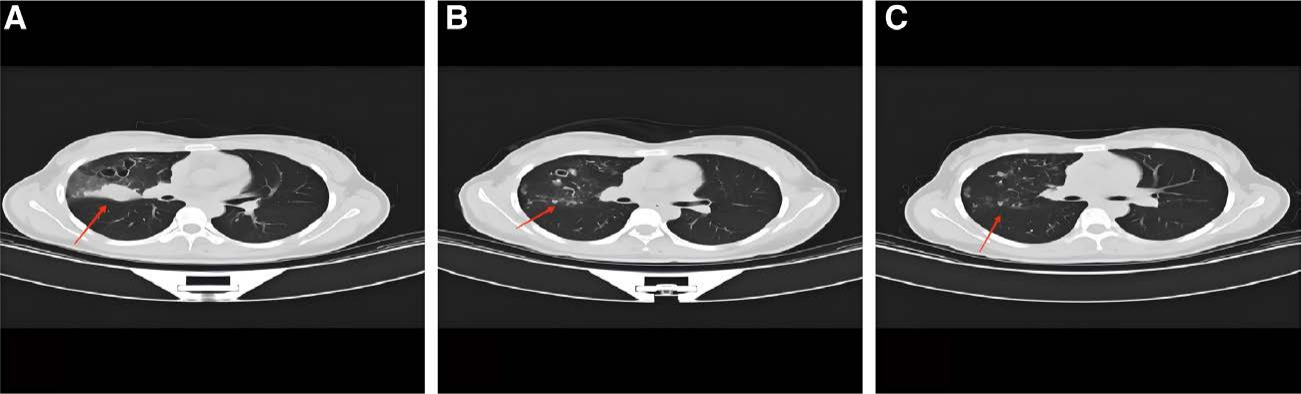
Serial chest computed tomography (CT) manifestations of pulmonary paragonimiasis before and after treatment. (A) Chest CT on September 9, 2022 (admission): patchy, nodular, and striated opacities in the right upper and middle lobes, with heterogeneous density, ill-defined margins, localized pleural adhesion, and multiple cavitary lesions. (B) Chest CT on November 26, 2022 (post-praziquantel treatment): most right lung lesions showed calcification; cystic lucencies were noted in the right middle lobe, with a significant reduction in lesion extent compared to (A). (C) Chest CT on February 13, 2023 (6-month follow-up): complete closure of the cavitary lesion observed in (A). CT = computerized tomography.

Upon admission, vital signs were as follows: temperature 37.5°C, pulse rate 70 bpm, respiratory rate 18 breaths/min, blood pressure 109/72 mm Hg (1 mm Hg = 0.133 kPa), and oxygen saturation 96% (normal range: 94–98%). The patient had a poor general condition but clear mental status, with no skin rash; bilateral lung respiratory sounds were slightly coarse without distinct dry or wet rales. Laboratory findings included: peripheral blood leukocyte count 6.19 × 10^9^/L (normal: 3.5–9.5 × 10^9^/L), total red blood cell count 4.5 × 10^12^/L (normal: 3.5–5.5 × 10^12^/L), platelet count 266 × 10^9^/L (normal: 125–350 × 10^9^/L), eosinophil count 0.48 × 10^9^/L (normal: 0.00–0.50 × 10^9^/L), and eosinophil percentage 7.8% (normal: 0.5–5.0%). A repeat chest CT on September 9, 2022 showed patchy, nodular, and striated shadows with heterogeneous density and ill-defined margins in the right upper and middle lobes, accompanied by localized pleural adhesion (Fig. 1A). Notably, initial chest imaging (Fig. 1A) revealed characteristic cavitary lesions of pulmonary paragonimiasis, which were initially misdiagnosed as pulmonary tuberculosis due to overlapping radiological features.

Given the persistent fever, cough, hemoptysis, and out-of-hospital test results, temporary diagnostic antituberculosis treatment was initiated: rifampicin 0.6 g/d (once daily), isoniazid 0.3 g/d (once daily), ethambutol 0.75 g/d (once daily), and pyrazinamide 0.5 g/d (3 times daily). Fiberoptic bronchoscopy on September 20, 2022 showed mild atrophy and scarring of bilateral bronchial mucosa. On September 29, 2022, bronchoalveolar lavage fluid (BALF) GeneXpert MTB/RIF test, sputum/BALF *Mycobacterium tuberculosis* RNA/DNA detection, and BACTEC MGIT 960 liquid culture were all negative. Considering the patient’s young age, lack of tuberculosis exposure history, ineffective out-of-hospital antituberculosis treatment, and multiple negative in-hospital tuberculosis-related tests, tuberculosis was excluded (exclusion criteria: negative sputum smear and BALF *M tuberculosis* culture; negative BALF GeneXpert MTB/RIF and tuberculosis RNA/DNA tests; and no typical tuberculous pathological changes such as caseous necrosis on bronchoscopic biopsy). BALF was then sent for metagenomic next-generation sequencing (mNGS), which revealed 87 Paragonimus sequences (BGI genomics)^[7]^ on October 6, 2022; Paragonimus antibody serological test (enzyme-linked immunosorbent assay [ELISA]) was positive. Follow-up history taking confirmed the patient had consumed raw crabs several years prior, leading to a revised diagnosis of paragonimiasis.

Due to the pharmacological interaction between rifampicin and praziquantel, antituberculosis treatment was suspended for 4 weeks, followed by oral praziquantel (1.2 g 3 times daily) for 3 consecutive days. A repeat blood test on November 25, 2022 showed total leukocyte count 4.45 × 10^9^/L, eosinophil count 0.16 × 10^9^/L, and eosinophil percentage 3.6%; liver/kidney function, coagulation parameters, and electrolytes were normal, and hemoptysis had resolved. Abdominal ultrasound revealed a calcified spot in the right hepatic lobe, parasplenic changes, and no abnormalities in the biliary tract, pancreas, or kidneys. Chest CT on November 26, 2022 showed most right lung lesions were calcified, with a cystic lucencies in the right middle lobe and significantly reduced lesion extent compared to prior imaging (Fig. 1B). After standard praziquantel therapy, follow-up CT (Fig. 1B) showed prominent calcification of lesions, indicating effective parasiticidal response. The patient received another 3-day course of oral praziquantel (1.2 g 3 times daily) before discharge. A follow-up chest CT on February 13, 2023 showed complete cavitary closure (Fig. 1C), and at the 6-month follow-up, complete resolution of cavitary lesions (Fig. 1C) confirmed the long-term efficacy of the treatment regimen with no recurrence.

### 2.1. Patient perspective (per CARE guidelines)

Following praziquantel administration, the persistent cough and hemoptysis that had afflicted me for 4 years resolved within 2 weeks, enabling the resumption of daily activities – including walking and studying – without fatigue. I strictly complied with the clinical recommendation to discontinue antituberculosis medications for a 4-week washout period prior to initiating praziquantel therapy, and completed the full treatment course as prescribed with no adverse reactions observed. Prior to this illness, I was unaware of the potential health hazards associated with consuming raw crabs. I am therefore grateful for the accurate diagnosis facilitated by mNGS. At the 6-month follow-up, I have maintained strict adherence to a diet of thoroughly cooked foods and regular health screening protocols, and I will indefinitely refrain from consuming raw crustaceans to prevent the recurrence of similar health conditions.

## 3. Discussion

### 3.1. Epidemiological features of paragonimiasis

Paragonimiasis is a food-borne zoonosis primarily transmitted via the ingestion of raw or undercooked freshwater crustaceans. In China, *Paragonimus westermani, Paragonimus heterotremus*, and *Paragonimus skrjabini* are the main pathogenic species,^[8]^ and Yunnan Province serves as a key endemic region owing to the local dietary practice of consuming raw stream crabs.^[9]^A notable epidemiological shift – spread from rural to urban areas – requires increased clinical vigilance.

For clinicians practicing in endemic regions, a detailed inquiry into the history of raw freshwater crustacean ingestion is paramount, particularly for patients with chronic respiratory symptoms (e.g., persistent cough, hemoptysis) that fail to respond to standardized antituberculosis therapy. The overlapping clinical and imaging manifestations between pulmonary paragonimiasis and pulmonary tuberculosis (e.g., hemoptysis, cavitary lesions) are the key primary driver of misdiagnosis, particularly in areas with a high prevalence of pulmonary tuberculosis such as Southwest China.^[10,11]^

### 3.2. Core difficulties in differentiating pulmonary paragonimiasis from pulmonary tuberculosis

Traditional tuberculosis tests (e.g., tuberculin pure protein derivative, specificity 77.3%)^[12]^ are prone to false-positive results, contributing to ~23.9% of paragonimiasis cases being misdiagnosed as tuberculosis^[13]^ – a pattern consistent with our patient’s initial misdiagnosis. Additionally, eosinophilia (>10%), a valuable auxiliary indicator for acute-phase paragonimiasis, loses diagnostic utility in the chronic phase (evidenced by our patient’s mild 7.8% elevation),^[13]^ and laboratory markers associated with pulmonary paragonimiasis (e.g., eosinophilia, elevated lactate dehydrogenase)^[14]^ lack absolute specificity, as they may overlap with findings of other infectious or inflammatory conditions.

Thus, accurate diagnosis requires the integration of epidemiological history (e.g., raw freshwater crustacean ingestion), serological testing, and imaging. As summarized in Table 1, the key differential diagnostic features distinguish the 2 diseases: pulmonary tuberculosis is characterized by tuberculosis exposure history, cavitary lesions with satellite nodules, and positive acid-fast bacilli detection, while pulmonary paragonimiasis is identified by raw freshwater crustacean ingestion history, eosinophilia, and positive Paragonimus antibody or egg detection. Notably, epidemiological history and serological testing are the most reliable differential markers, whereas exclusive reliance on imaging features risks misdiagnosis due to their substantial overlap between the 2 diseases.

**Table 1:** Key differential diagnostic features between pulmonary paragonimiasis and pulmonary tuberculosis.

Disease type	Pulmonary tuberculosis	Pulmonary paragonimiasis
Epidemiological history	Tuberculosis exposure history (e.g., contact with active TB patients)	Raw freshwater crustacean ingestion (e.g., raw stream crabs, crayfish)
Clinical symptoms	Low-grade fever, fatigue, emaciation, night sweats, expectoration, sputum, hemoptysis, chest pain, dyspnea	Early stage: abdominal pain, diarrhea, anorexia; late stage: chest pain, dyspnea, chronic cough, fruit-jelly-like sputum (Paragonimus eggs detectable in sputum)
Image findings	1. Lesion location: apical posterior segment of upper lobes, posterior/posterior basal segments of lower lobes 2. Core features: multiple cavitary lesions (thin-walled, with satellite nodules), bronchial dissemination, pleural effusion/adhesion	1. Early stage: increased, thickened, reticular lung markings 2. Late stage: irregular nodular opacities (blurred edges), pleural thickening/adhesion, focal calcification, mediastinal lymphadenopathy, cavitary lesions (thick, irregular walls)
Laboratory examinations	1. Sputum acid-fast bacilli (AFB) smear/culture(+) 2. Specific antituberculosis antibody(+) 3. *Mycobacterium tuberculosis* DNA/RNA detection(+) 4. Tuberculin pure protein derivative (PPD) test: often strongly positive (low specificity)	1. Egg detection (stool, sputum, pleural effusion, or necrotic tissue): gold standard(+) 2. Serological tests: paragonimus antibody ELISA(+), intradermal test(+) 3. Peripheral blood: eosinophilia (>10% in acute phase, 5–10% in chronic phase) 4. Tissue biopsy: detection of Paragonimus larvae/adults (for suspected cases)

AFB = acid-fast bacilli, ELISA = enzyme-linked immunosorbent assay, PPD = pure protein derivative, TB = tuberculosis.

Given the overlapping manifestations, false-positive traditional tests, and stage-dependent indicators, a stratified diagnostic approach is essential to improve accuracy.

### 3.3. Diagnostic strategy: stratified selection and clinical value of mNGS

Paragonimiasis diagnosis should employ a “first-line screening + difficult case correction” approach to balance diagnostic accuracy and cost-effectiveness across diverse medical resource settings:

First-line screening (resource-limited areas): serum Paragonimus antibody ELISA (sensitivity: 92%, specificity: >90%^[15]^) combined with chest CT (focusing on reticular markings + nodular opacities) represents the most cost-effective strategy, covering the majority of suspected cases. Traditional egg detection (in sputum, BALF, etc) exhibits low sensitivity (detection rate: 11.7%^[16]^) and is not recommended as the sole diagnostic basis.

Difficult case correction: mNGS effectively detects rare pathogens (e.g., 87 Paragonimus sequences in the patient’s BALF) without interference from atypical indicators (e.g., mild eosinophilia), emerging as a key tool for correcting misdiagnosis.^[17-20]^ In resource-limited areas, however, serological screening should be prioritized for high-risk populations (eosinophilia + raw freshwater crustacean ingestion history) rather than the indiscriminate use of mNGS.

### 3.4. Treatment focus: drug interactions and safety monitoring

The World Health Organization (WHO) designates praziquantel as the first-line agent for paragonimiasis, with a standardized dosage of 25 mg/kg administered 3 times daily for 3 consecutive days per course; typically 2 to 3 courses are required for optimal outcomes. If disease recurrence, praziquantel resistance, or patient intolerance to praziquantel occurs, triclabendazole (10 mg/kg, oral twice) can be used as an alternative, with better tolerance than praziquantel.^[21]^

Rifampicin, a core antituberculosis agent, is a potent CYP3A4 inducer that markedly accelerates the hepatic metabolism of praziquantel, lowering its plasma concentration below the therapeutic threshold.^[22]^ Pharmacokinetic studies show that rifampicin initiates enzyme induction within hours of oral administration, reaches peak induction after ~2 weeks, and its inductive effect persists for ~2 weeks after discontinuation (total cycle: ~4 weeks).^[22]^ Therefore, antituberculosis regimens containing rifampicin must be discontinued for a 4-week washout period before initiating praziquantel treatment – this approach resulted in lesion calcification and cavity closure in the present case, validating its clinical imperative. Close monitoring of adverse reactions (gastrointestinal symptoms, cardiac dysfunction) is required during praziquantel treatment.^[23]^

## 4. Conclusion

This case emphasizes that overlapping clinical and imaging manifestations between pulmonary paragonimiasis and pulmonary tuberculosis are the primary causes of misdiagnosis. Three key clinical measures can improve diagnostic accuracy and patient prognosis:

Systematic documentation of raw freshwater crustacean ingestion history among patients in endemic regions:

A stratified diagnostic approach – initial screening with Paragonimus antibody ELISA combined with chest CT, and mNGS reserved for cases unresponsive to antituberculosis therapy;
Standardized management of drug–drug interactions – a 4-week washout period following rifampicin discontinuation prior to praziquantel initiation.

Clinicians practicing in paragonimiasis-endemic regions (e.g., Southwest China) should incorporate paragonimiasis into the differential diagnosis of chronic respiratory conditions. Proactive documentation of epidemiological history and integration of multi-modal diagnostic tools mitigate unnecessary antituberculosis treatment and drug-related toxicity, thereby optimizing patient outcomes.

## Author contributions

**Conceptualization:** Rong Li, Zhong-Ping Bai, Gao Song, Cai-Qiong Zhang.

**Data curation:** Rong Li, Zhong-Ping Bai, Gao Song, Cai-Qiong Zhang.

**Formal analysis:** Rong Li, Zhong-Ping Bai, Xing Liu.

**Funding acquisition:** Zhong-Ping Bai, Cai-Qiong Zhang, Wei-Xian Li, Xing Liu.

**Investigation:** Rong Li, Wei-Xian Li, Xing Liu.

**Methodology:** Ling-Jun Shen, Wei-Xian Li, Xing Liu.

**Project administration:** Rong Li, Zhong-Ping Bai, Ling-Jun Shen, Wei-Xian Li, Xing Liu.

**Resources:** Rong Li, Zhong-Ping Bai, Ling-Jun Shen, Wei-Xian Li.

**Software:** Rong Li, Zhong-Ping Bai, Cai-Qiong Zhang, Xing Liu.

**Supervision:** Zhong-Ping Bai, Gao Song, Cai-Qiong Zhang, Ling-Jun Shen, Xing Liu.

**Validation:** Rong Li, Zhong-Ping Bai, Gao Song, Xing Liu.

**Visualization:** Rong Li, Gao Song, Ling-Jun Shen.

**Writing – review & editing:** Rong Li, Gao Song.

**Writing – original draft:** Gao Song, Xing Liu.
